# 162. Who’s Being Missed? Baseline Evaluation of Colorectal Cancer Screening in a Ryan White Population

**DOI:** 10.1093/ofid/ofaf695.057

**Published:** 2026-01-11

**Authors:** Emily Dyer, Jennifer M Davis, Sara H Bares, Elizabeth Lyden, Nichole N Regan

**Affiliations:** Univeristy fo Nebraska Medical Center, Omaha, NE; University of Nebraska Medical Center, Omaha, NE; University of Nebraska Medical Center, Omaha, NE; University of Nebraska Medical Center, Omaha, NE; University of Nebraska Medical Center, Omaha, NE

## Abstract

**Background:**

Cancer is the leading cause of death among people with HIV (PWH). Estimates of colorectal cancer (CRC) screening rates among PWH vary widely (25%-86%), with some studies reporting higher rates of screening than the general population (63.5% national CRC screening average in 2023). We assessed CRC screening rates at our Midwestern Ryan White Clinic and identified factors associated with missed opportunities for screening.

**Table 1:**
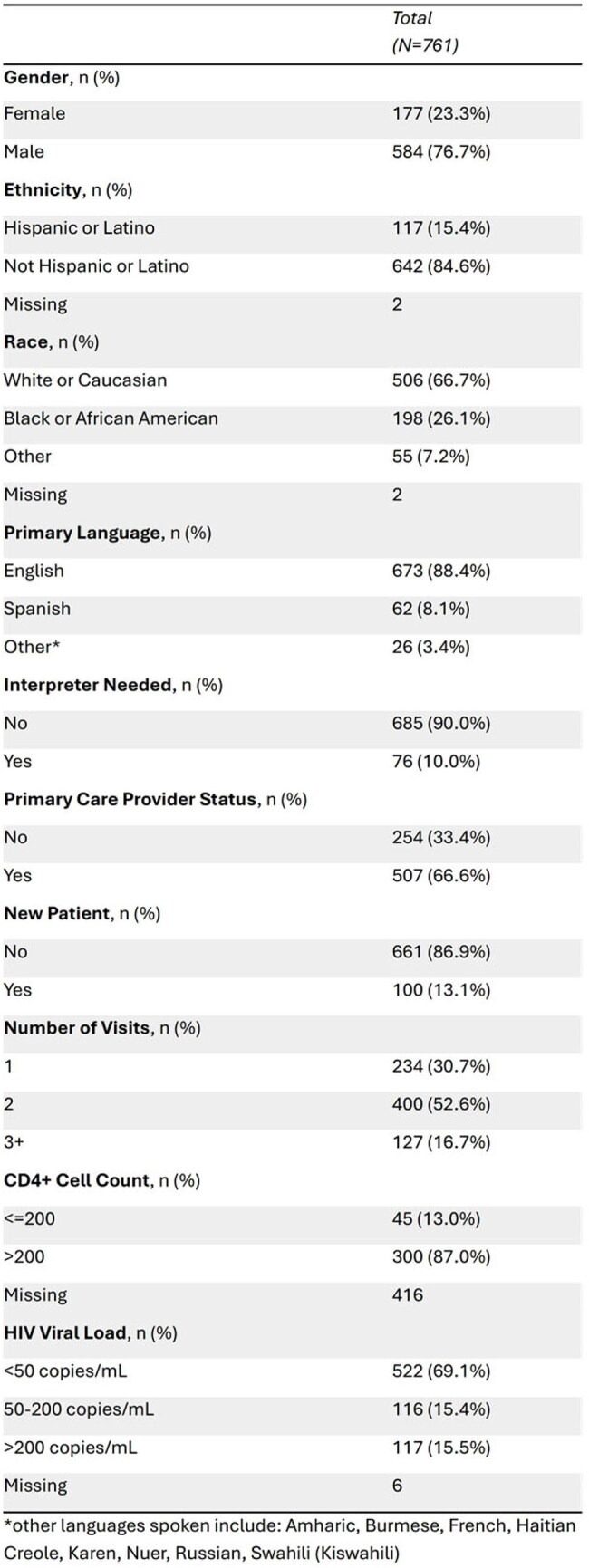
Patient Characteristics

**Table 2:**
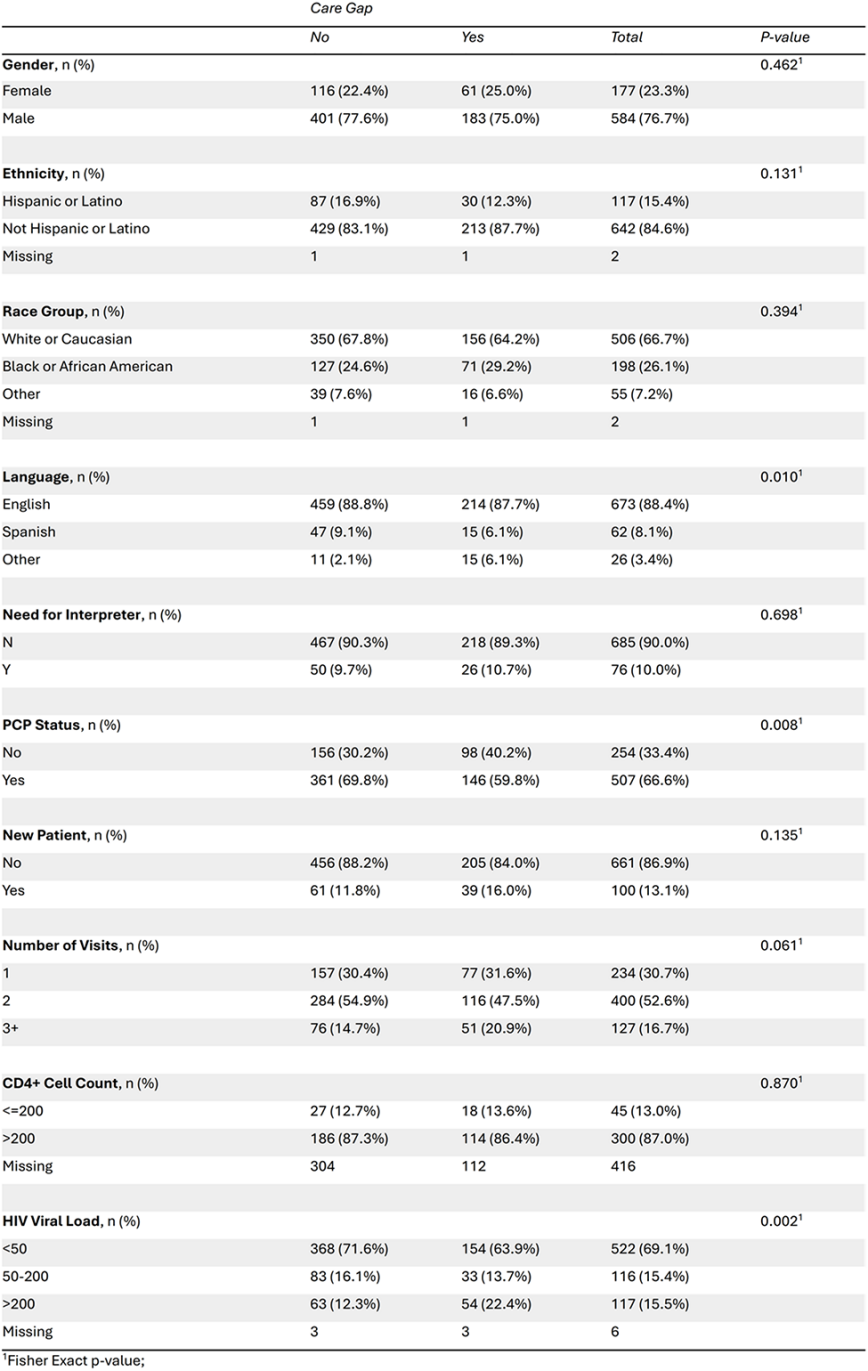
Comparisons of Demographics with Presence or Absence of Care Gap

**Methods:**

We conducted a retrospective review of PWH ≥ 45 years old evaluated in clinic between July 1, 2023 to June 30, 2024. Patient need for CRC screening was measured by the presence of a “care gap” in the electronic medical record during the study year. Demographic and clinical data, including type of CRC screening ordered, were extracted. Descriptive statistics summarized patient characteristics. Fisher’s exact test assessed associations between patient characteristics and CRC screening outcomes. All analyses used SAS v9.4 (p < 0.05 considered significant).

**Table 3:**
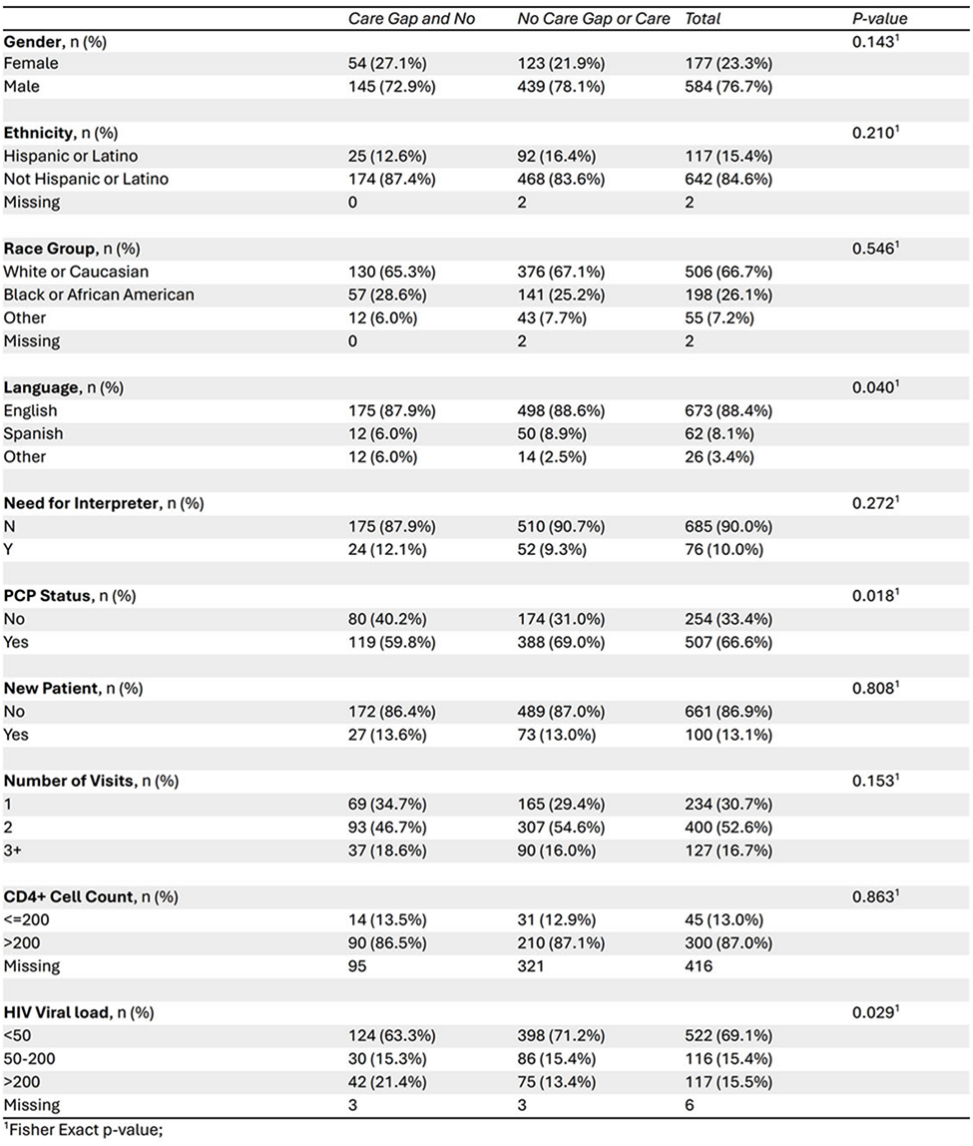
Comparisons of Demographics of Those with a Care Gap and No CRC Screening Ordered

**Results:**

Among 761 patients (median age 56), 66.7% were White, 26.1% Black, and 15.4% Hispanic or Latino. (Table 1) A care gap was present in 244 (32.1%), of whom 82% (199/244) had no CRC screening ordered. (Table 2) Variables that showed a statistically significant association with both a care gap1 and lack of orders2 were speaking a primary language other than English (p=0.011; p= 0.042), not having a primary care provider (PCP) (p= 0.0081; p=0.0182), and having a viral load >200 copies/mL (p= 0.0021; p= 0.0292). (Tables 2 & 3)

**Conclusion:**

CRC screening rates in our Ryan White clinic exceed national averages, but key disparities persist. Patients with limited English proficiency, no primary care engagement, and HIV viremia were more likely to have missed opportunities for screening. These data will inform future quality improvement initiatives including tailored interventions for those with limited English proficiency, increased linkage to primary care, and care coordination for those with HIV viremia.

**Disclosures:**

Jennifer M. Davis, MD, GSK/ViiV Healthcare: Grant/Research Support Sara H. Bares, MD, Gilead Sciences: Expert Testimony

